# Skeletal Dysplasias Caused by Sulfation Defects

**DOI:** 10.3390/ijms21082710

**Published:** 2020-04-14

**Authors:** Chiara Paganini, Chiara Gramegna Tota, Andrea Superti-Furga, Antonio Rossi

**Affiliations:** 1 Department of Molecular Medicine, Unit of Biochemistry, University of Pavia, 27100 Pavia, Italy; chiara.paganini01@universitadipavia.it (C.P.); chiara.gramegnatota01@universitadipavia.it (C.G.T.); 2 Division of Genetic Medicine, Lausanne University Hospital, University of Lausanne, 1011 Lausanne, Switzerland; asuperti@unil.ch

**Keywords:** sulfate metabolism, sulfotransferase, glycosaminoglycan, proteoglycan, cartilage, genotype phenotype correlation, skeletal disorders

## Abstract

Proteoglycans (PGs) are macromolecules present on the cell surface and in the extracellular matrix that confer specific mechanical, biochemical, and physical properties to tissues. Sulfate groups present on glycosaminoglycans, linear polysaccharide chains attached to PG core proteins, are fundamental for correct PG functions. Indeed, through the negative charge of sulfate groups, PGs interact with extracellular matrix molecules and bind growth factors regulating tissue structure and cell behavior. The maintenance of correct sulfate metabolism is important in tissue development and function, particularly in cartilage where PGs are fundamental and abundant components of the extracellular matrix. In chondrocytes, the main sulfate source is the extracellular space, then sulfate is taken up and activated in the cytosol to the universal sulfate donor to be used in sulfotransferase reactions. Alteration in each step of sulfate metabolism can affect macromolecular sulfation, leading to the onset of diseases that affect mainly cartilage and bone. This review presents a panoramic view of skeletal dysplasias caused by mutations in genes encoding for transporters or enzymes involved in macromolecular sulfation. Future research in this field will contribute to the understanding of the disease pathogenesis, allowing the development of targeted therapies aimed at alleviating, preventing, or modifying the disease progression.

## 1. Introduction

Sulfated compounds include a wide array of substances, ranging in molecular weight from less than 10^3^ Da to greater than 10^6^ Da, that undergo striking changes in their physicochemical properties upon the addition of the negatively charged sulfated groups. Sulfation increases electrostatic interactions, water solubility, converts lipophilic molecules to amphiphiles, and can lead to conformational changes both in low and high-molecular-weight molecules. Whereas phosphorylation is central to intracellular signal transduction, sulfation modulates cell–cell and cell–matrix communication.

The highest density of sulfate groups is found in proteoglycans (PGs), a broad category of macromolecules characterized by a core protein to which one to over one hundred glycosaminoglycan (GAG) chains are attached [1]. PGs are present in the cell nucleus, in cytoplasm, and at the cell membranes, but they are particularly abundant in the extracellular matrix (ECM) of connective tissues. The highly acidic and hydrophilic properties of GAGs have a major influence on tissue hydration, elasticity, and cation composition. Furthermore, they bind with high-affinity ECM proteins, growth factors, enzymes, and cell surface receptors [2,3,4].

Aside from PGs, many other molecules can be sulfated showing different effects; for instance, sulfate moieties added to hormones influence their biological activity [5]. Sulfoglycolipids such as sphingolipids and galactoglycerolipids are abundant in myelin as well as spermatozoa, kidney, and small intestine [6]. Sulfation of tyrosine residues represents the prevalent posttranslational modification of many secretory and membrane proteins and peptides that may significantly influence their function [7]. Sulfation also has a significant role in the biotransformation of many endogenous low-molecular-weight compounds including catecholamines and iodothyronines [8], cholesterol, bile acids, and steroids [9,10,11].

These examples highlight that proper sulfation of endogenous molecules is a widespread phenomenon so far poorly explored, although it is essential for growth and development. Indeed several heritable disorders caused by defects in the biosynthesis and catabolism of sulfated compounds are associated mainly with abnormal development of the skeleton [12].

Skeletal dysplasias are a huge and heterogeneous group of genetic disorders that affect mainly, but not exclusively, cartilage and bone. Beyond orthopedic complications, neurological, auditory, visual, cardiac, pulmonary, and renal problems could be present because the causative genes might also have functional roles in tissues other than the skeleton [13,14]. Nowadays 461 disorders have been characterized based on clinical, radiographic and molecular observations and 437 disease genes have been identified [15]. Skeletal dysplasias show a wide range of severity from very mild phenotypes compatible with life to fetal and neonatal lethality. Interestingly, mutations in different genes may cause the same skeletal dysplasia or mutations in the same gene are related to different skeletal disorders [14]. Genes involved in skeletal dysplasias encode for different protein products including ECM proteins, enzymes, cellular transporters, transcription factors, signal transducers, channel proteins, chaperones, cytoplasmic proteins, cilia, proteins involved in cell cycle, and chromatin modifying enzymes [13,14].

This review deals with disorders of the biosynthesis of sulfated macromolecules, which are associated mainly with abnormal development of the skeleton.

## 2. The Cellular Metabolism of Sulfate

**Figure 1:**
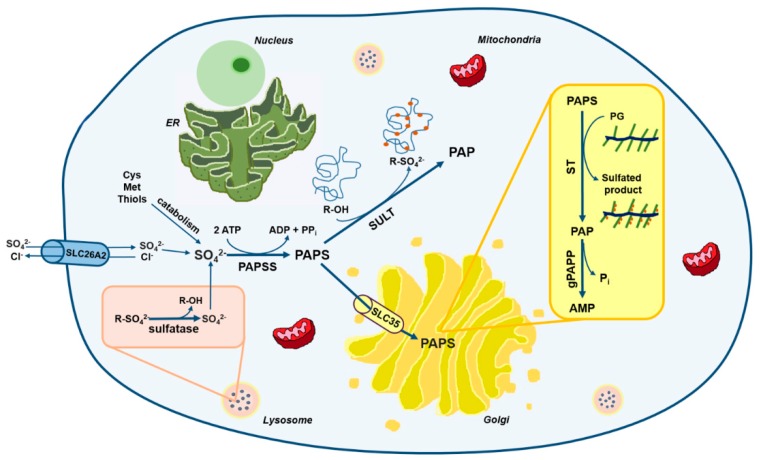
Schematic view of sulfate metabolism in cells. Intracellular level of sulfate depends mainly on extracellular uptake through membrane transporters such as the solute carrier family 26 member 2 (SLC26A2). A small amount of sulfate comes also from the catabolism of sulfur-containing amino acids and thiols or from sulfatase reactions in lysosomes. Once in cell, sulfate is activated to 3′-phosphoadenosine 5′-phosphosulfate (PAPS) by PAPS synthetase (PAPSS). PAPS represents the universal sulfate donor and is used by cytosolic sulfotransferases (SULTs) for hormone and xenobiotics sulfation or by Golgi sulfotransferases (STs) for the sulfation of macromolecules such as PGs. The solute carrier family 35 member B2 (SLC35B2) and SLC35B3 transporters allow Golgi uptake of PAPS. During sulfotransferase reactions, phosphoadenosine phosphate (PAP) is produced as a by-product and is hydrolyzed to AMP and phosphate by a Golgi resident phosphoadenosine phosphate phosphatase (gPAPP, also known as IMPAD1 or BPNT2).

Sulfation is mechanistically analogous to phosphorylation: in analogy to ATP, the phosphate donor used by several kinases, there is a “universal” sulfate donor for different substrate specific sulfotransferases. Studies in prokaryotes, yeast, liver, and cartilage tissue have demonstrated that the universal sulfate donor for sulfotransferase reactions is 3′-phosphoadenosine 5′-phosphosulfate (PAPS) [16,17,18]. Thus, in all mammalian tissues, intracellular sulfate is activated to PAPS through the sulfate activation pathway in order to be used in sulfation reactions catalyzed by sulfotransferases (Figure 1) [19,20]. 

### 2.1. The Origin of Intracellular Sulfate

Endogenous sulfate is essential for PAPS synthesis. The cytoplasmic sulfate pool may be either derived from the extracellular fluids or from the catabolism of sulphur containing amino acids and other thiols with production of sulfite, which is then oxidized to sulfate. Intracellular sulfate can also come from the degradation of sulfated molecules through sulfatases [21]. 

In healthy adults, one third of sulfate is provided from the diet [22]; sulfate is adsorbed in the small intestine and the plasma level, that in humans span a broad range (250–400 µM), is maintained by renal reabsorption in proximal tubule through SLC13A1 and SLC26A1 transporters [21,23]. Age [24], gestational period [25] and diseases [26] alter sulfate homeostasis affecting sulfate concentrations in body fluids [22].

The transport of sulfate ions across cell membranes and epithelial tissues is mediated by carrier proteins. Sulfate transporters function either as sulfate/chloride antiporters or sodium/sulfate symporters [21,27,28,29]. Human sulfate transporters belong to different solute linked carrier families such as SLC4, SLC13 and SLC26. Transporters of the same family, such as the SLC26 transporter family, show similar structure and sequence, but mutations in each gene cause different phenotypes due to different tissue distribution and exchanged anion [30]. For instance, SLC26A1 and SLC26A2 are both present in kidney and intestine, but the former is primarily expressed in hepatocytes, while the latter in chondrocytes [31]. In the SLC13 family, SLC13A1 is mainly expressed in renal tissue [32], while SLC13A4 in placenta [33]. 

Among sulfate transporters, SLC26A2 is also known as the diastrophic dysplasia sulfate transporter (DTDST). The identification of this sulfate/chloride antiporter was the end result of a long-term project aimed at elucidating the molecular basis of diastrophic dysplasia (DTD), a skeletal dysplasia particularly frequent in the Finnish population [34]. The hypothesis that DTD was associated with impaired sulfate transport was confirmed by the reduced sulfate uptake in cultured fibroblasts from patients and by the identification of mutations in the candidate gene in these individuals [34]. 

Despite the wide distribution of sulfate transporters in different organs, not all tissues use inorganic sulfate as the main sulfate source. Kidney cells also use cysteine, N-acetylcysteine, and glutathione, whereas in lung cells cysteine is the most efficient source of sulfate [35]. In human fibroblast cultures, at less than physiological extracellular sulfate concentrations, cysteine is a major contributor to the intracellular sulfate pool [36]. 

A small fraction of intracellular sulfate can come from lysosomal degradation of the carbohydrate moiety of glycoproteins and GAGs that produces monosaccharides and sulfate which must efflux from the lysosomes before re-entering biosynthetic pathways [37].

In conclusion, based on our knowledge it is likely that, the three distinct sources of sulfate work together to maintain the intracellular sulfate pool, but with different prevalence in different cell types. 

### 2.2. The Sulfate Activation Pathway 

Sulfation reactions require PAPS as universal sulfate donor. Its concentration differs among tissues and species; in humans it ranges between 3.6–22.6 nmol/g tissue [16].

The activation of sulfate to PAPS results from the concerted action of two enzymes ATP sulfurylase and adenosine 5′-phosphosulfate (APS) kinase which are located on separate polypeptide chains in bacteria, fungi, yeast, and plants, while in animals they are present in a bifunctional protein named PAPS synthetase (PAPSS) [38]. The catalytic domain of APS kinase is located in the amino-terminal region, whereas the ATP sulfurylase domain is in the carboxy-terminal part. The first step is catalyzed by ATP sulfurylase and involves the activation of inorganic sulfate with ATP to form APS and pyrophosphate. The second step, the conversion of APS and another molecule of ATP in PAPS and ADP, is catalyzed by APS kinase. The first reaction is not energetically favoured and it is the rate limiting step in PAPS formation; however, subsequent hydrolysis of pyrophosphate and the rapid utilization of APS in the second reaction relieve the energy constraint of the overall reaction. PAPSS exists as two isozymes encoded by genes located on separate chromosomes. The human gene for PAPS synthetase 1 (PAPSS1) is located on chromosome 4q25–26 [39]; while the gene for human PAPS synthetase 2 (PAPSS2) has been localized to chromosome 10q23–24 [40]. Interestingly the mouse and human PAPSS2 isozymes were discovered through investigation of specific developmental dwarfism disorders, i.e., brachymorphism in mice and a form of spondyloepimetaphyseal dysplasia in humans [40,41]. More recently, PAPSS2 has also been implicated in steroid metabolism [42], while mutations in PAPSS1 have not been linked to any human pathology suggesting embryonic lethality since PAPSS1 is the main isoform in developing nervous system [21,43].

In both human and mouse species, PAPSS1 and 2 are 76% identical [20], but the catalytic activity of the PAPSS2 variant is 10- to 15-fold higher than that of PAPSS1 [44]. The two isoforms have different cellular localization: PAPSS1 localizes mainly to the nucleus, while PAPSS2 to the cytosol [45]. Moreover the two isoenzymes differ also for tissue localization, since PAPSS1 is expressed in skin and brain, whereas PAPSS2 in liver and cartilage [20]. 

PAPS is the universal sulfate donor for sulfation either in the cytosol or in the Golgi. In the cytosol PAPS is used for sulfation of a wide variety of endogenous compounds including hormones and neurotransmitters as well as drugs and xenobiotics. On the other hand, sulfation of carbohydrates, peptides and proteins occurs in the Golgi; for this purpose PAPS is transported to the Golgi by specific PAPS transporters (PAPSTs). Two different PAPST isoforms have been cloned, PAPST1 and PAPST2 [46,47], also known as SLC35B2 and SLC35B3, respectively [48,49]. The two transporters are closely related and show similar transport activity; they are both widely expressed among tissues, in particular PAPST2 is highly expressed in colon [46]. Kinetic studies of PAPSTs have suggested that PAPS transport occurs through an antiport mechanism that might involve phosphoadenosine phosphate (PAP) or AMP [49]. So far, no human genetic disorders have been linked to mutations in *SLC35B2* and *SLC35B3* genes, but cartilage defects have been reported in *slc35b2* null zebrafish mutants [21,50].

### 2.3. The Sulfation Pathway

The transfer of sulfate from the activated donor, PAPS, to a variety of molecules is catalyzed by sulfotransferases. Two classes of sulfotransferases are present in eukaryotes: cytosolic sulfotransferases, that sulfate small molecules such as hormones, amines and xenobiotics including drugs, and Golgi resident sulfotransferases that sulfate larger substrates involved in the secretory pathway [43,51]. 

In human genome 13 genes for cytosolic sulfotransferases (SULTs) exist and these enzymes are classified in four families (SULT1, SULT2, SULT4 and SULT6) based on distribution and substrate preference, even if SULTs may show overlapping substrate specificity [52]. SULT enzymes, with the exception of SULT6, are soluble cytosolic proteins that form dimers thank to a 10 amino acid sequence at the C-terminal end named KTVE motif [48,53]. 

Carbohydrate sulfotransferases (STs) are membrane bound enzymes present in the Golgi; up to now, 37 genes have been identified in humans [21]. STs are stereoselective and exhibit strict substrate specificity [2]; the addition of a sulfate group can convert a common carbohydrate structural motif into a unique recognition site for a specific receptor. Members of ST families show similar primary structure and identical substrate specificity, but they are differently distributed [2].

In the Golgi also specific tyrosine residues of secreted proteins and peptides are sulfated by tyrosylprotein sulfotransferases (TPST1 and TPST2) [54]. These two enzymes show 67% amino acid identity and use PAPS as obligate sulfate donor. Tyrosine sulfation can determine the bioactivity of several neuropeptides or expand the functions of some proteins. Moreover, sulfated tyrosines are important to boost protein-protein or protein-biopolymer interactions [48,55].

PAPS-dependent sulfotransferase reactions produce PAP as by-product that can inhibit, via negative feedback, sulfotransferases [48]. To prevent their inhibition, rapid degradation of PAP in AMP and phosphate by a PAP phosphatase is important as demonstrated in chondrodysplasia with joint dislocations gPAPP type caused by mutations in the *IMPAD1* gene (recently renamed *BPNT2*, 3′(2′), 5′-bisphosphate nucleotidase 2), encoding for a Golgi resident PAP phosphatase (gPAPP) or in the gene trap knock-out mice for the same gene [56,57,58].

Sulfatases, hydrolytic enzymes belonging to the alkaline phosphatase superfamily [48], play an opposite role to that of sulfotransferases. Indeed, these enzymes are a highly conserved family of proteins that catalyze the hydrolysis of sulfate ester bonds from a wide variety of substrates including GAGs, sulfolipids and steroid sulfates [59]. Seventeen genes have been identified in the human genome [21] encoding sulfatase enzymes, that share similar size, high glycosylation level, sequence homology and similar active site. Based on their subcellular localization, sulfatases are grouped in two main categories: those expressed in lysosomes, that act at acidic pH and are involved in catabolism, and those present in the endoplasmic reticulum, Golgi apparatus and at the cell surface acting at neutral pH and more likely involved in biosynthetic, rather than catabolic, pathways [59].

## 3. Sulfate Metabolism and Genetic Diseases 

**Figure 2:**
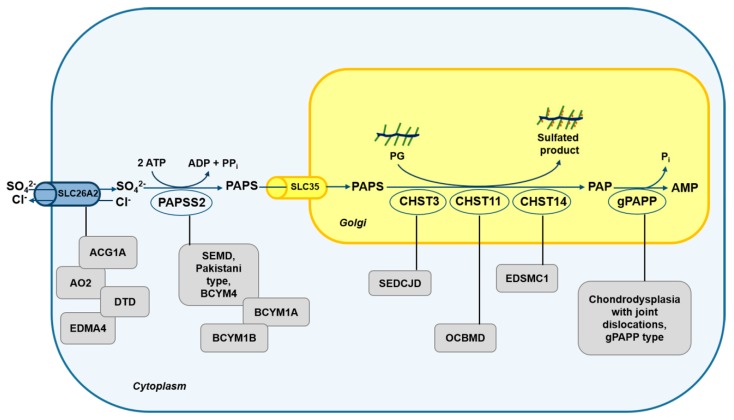
Schematic diagram of the defects in the sulfation pathway causing skeletal dysplasias. Mutations in genes encoding for transporter and enzymes involved in sulfate metabolism cause several skeletal dysplasias (grey box). ACG1B, achondrogenesis type 1B; AO2, atelosteogenesis type 2; DTD, diastrophic dysplasia; EDM4, recessive multiple epiphyseal dysplasia; SEMD, spondyloepimetaphyseal dysplasia; BCYM4, brachyolmia type 4; BCYM1A, brachyolmia type 1, Hobaek form; BCYM1B, brachyolmia type 1, Toledo form; SEDCJD, spondyloepiphyseal dysplasia with congenital joint dislocations; EDSMC1, Ehlers–Danlos syndrome musculocontractural type 1 and OCBMD, osteochondrodysplasia, brachydactyly and overlapping malformed digits.

Genetic defects have been identified in several steps of PG biosynthesis, leading to a huge number of diseases that primarily affect cartilage and endochondral bone [12]. The cartilage phenotype might be explained by the high amount of sulfated PGs that are synthesized and deposited in the cartilage ECM. Among these disorders, diseases involving sulfate uptake, metabolic activation, and sulfation of GAGs are discussed in this review (Figure 2).

### 3.1. Skeletal Dysplasias Linked to Proteins Involved in Sulfate Metabolism

*SLC26A2* linked chondrodysplasias represent a heterogeneous group of skeletal diseases caused by mutations in the *SLC26A2* gene encoding for a sulfate transporter present on the cell membrane, also known as diastrophic dysplasia sulfate transporter. There are currently four distinct phenotypes associated to *SLC26A2* mutations; this division is useful for differential diagnosis and prognosis, but has no biological basis since the spectrum of diseases based on clinical and radiological criteria is continuous. The four different conditions described in decreasing order of severity include: achondrogenesis type 1B (ACG1B, MIM 600972), atelosteogenesis type 2 (AO2, MIM 256050), diastrophic dysplasia (DTD, MIM 222600) and recessive multiple epiphyseal dysplasia (EDM4, MIM 226900) [60]. Key clinical features comprise lethality in the fetal period or immediately after birth (ACG1B and AO2), shortened limbs (all but not EDM4), joint pain, joint contractures, “hitchhiker” thumb (AO2 and DTD), cystic swelling of the external ear (DTD), cleft palate (DTD and AO2), scoliosis, clubfoot and double-layered patellae (EDM4). Two other conditions originally described as distinct lethal skeletal dysplasias based on radiographic findings, namely de la Chapelle dysplasia and McAllister dysplasia [61,62], were proven to be related to AO2 based on biochemical and molecular data [63,64].

In these disorders the different clinical phenotypes have been linked to the mutation severity, the residual activity of the sulfate transporter and the undersulfation level of cartilage PGs [60,65,66], but it appears that the nature of the *SLC26A2* mutations is not the only factor determining phenotypic severity. Phenotypic differences have been observed between unrelated and even first-degree relatives with the same mutation. In the dtd mouse, an animal model of human DTD, the skeletal phenotype has been confirmed by the presence of reduced skeletal growth, deformities of long bones, reduced toluidine blue staining of cartilage, chondrocytes of irregular size and delay in the formation of the secondary ossification center. Interestingly impaired sulfate uptake was demonstrated in chondrocytes, osteoblasts, and fibroblasts, but significant PG undersulfation was detected only in cartilage, despite the generalized sulfate uptake defect [67]. Studies on the growth plate of the animal model demonstrated reduced chondrocyte proliferation due to altered Indian hedgehog (Ihh) signalling [68] and reduced phosphorylation of the pocket protein p130 that inhibits transcription factors of the E2F family leading to the block in the G1 phase of cell cycle progression [69]. Even if sulfate uptake from the extracellular environment is crucial for PG sulfation in chondrocytes, an alternative source of sulfate, namely intracellular thiol compounds, has been reported in the dtd mouse suggesting potential pharmacological approaches to DTD [70]. Recently an over activation of fibroblast growth factor receptor 3 (FGFR3) signalling has been described in a *Slc26a2* knock-out mouse, an animal model of ACG1B and AO2. This finding suggests the involvement of altered FGFR3 pathway in the pathogenic mechanism contributing to the lethal phenotype [71].

*Spondyloepimetaphyseal dysplasia (SEMD), Pakistani type* has been described in a large family from Pakistan by Ahmad et al. in 1998 [72]. The clinical phenotype includes short stature, short and bowed lower limbs with enlarged knee joints, kyphoscoliosis, mild brachydactyly, delayed ossification of the epiphyses and osteoarthritis [72]. In these patients mutations in the *PAPSS2* gene, encoding for the 3’-phosphoadenosine-5’-phosphosulfate synthetase 2, have been identified [40,73]. PAPSS2 impairment causes reduced intracellular synthesis of PAPS leading to reduced macromolecular sulfation. SMED Pakistani type has been defined in OMIM as *Brachyolmia type 4* (BCYM4, MIM 612847), even if this name is obsolete and rarely used. Compound heterozygous mutations in *PAPSS2* have been identified in a girl with alteration of steroid metabolism as demonstrated by premature pubarche, hyperandrogenic anovulation, very low dehydroepiandrosterone sulfate ester (DHEAS) levels, and increased androgen levels [42]. This phenotype can be explained by the observation that PAPS is the sulfate donor not only for macromolecular sulfation, but also for steroid hormones as androgens. Indeed PAPS is required for the conversion of the androgen hormone precursor, dehydroepiandrosterone (DHEA), to its inactive sulfate ester (DHEAS), reducing its conversion to an active androgen. Interestingly, the bone phenotype does not present long bone epiphyseal or metaphyseal changes demonstrating that it is milder in this patient compared to SEMD Pakistani type.

*Brachyolmia type 1* including *Hobaek* and *Toledo forms* (BCYM1A and 1B, MIM 271530 and 271630, respectively) is characterised by short trunk, platyspondyly, irregular and narrow intervertebral spaces, scoliosis, precocious calcification of the costal cartilage and in some cases corneal opacities [74]. In the Toledo form of brachyolmia abnormal chondroitin sulfation has been described as demonstrated by chondroitin sulfate (CS) undersulfation in urine [74] and low activity of PAPS-chondroitin sulfate sulfotransferase in serum [75]. In 2012, *PAPSS2* mutations have been identified in brachyolmia type 1 patients [73], making this disorder allelic to the SEMD Pakistani type. Further studies involving 13 patients from 10 families identified *PAPSS2* mutations in brachyolmia type 1 and confirmed that brachyolmia type 1A, 1B and 4 are indeed the same disorder [76]. Despite PAPS involvement in androgen metabolism, only a minority of these patients showed signs of androgen excess or increased serum DHEA.

*Chondrodysplasia with joint dislocations, gPAPP type* (MIM 614078) is characterised by short stature, chondrodysplasia with brachydactyly, congenital joint dislocations, micrognathia, cleft palate and facial dysmorphism. It is caused by mutations in the *IMPAD1* gene recently renamed *BPNT2* encoding for gPAPP [56]. BPNT2 impairment reduces the hydrolysis of PAP, the by-product of the sulfotransferase reactions, leading to its accumulation in the Golgi and to the inhibition of sulfotransferase reactions via negative feedback. The final consequence of BPNT2 impairment is the undersulfation of macromolecules. Unfortunately, cells or cartilage biopsies from patients were not available, but through a gene trap approach two *Impad1* knock-out mice have been generated and are characterised by severe dwarfism, skeletal defects and abnormal joint formation. Even if they showed a perinatal lethal phenotype, the impaired CS and heparan sulfate (HS) proteoglycan sulfation confirmed that this condition is associated with defective synthesis of sulfated PGs. [57,58]. The overlapping features of chondrodysplasia with joint dislocations, gPAPP type with Catel-Manzke syndrome and Desbuquois dysplasia type 1, other skeletal dysplasias caused by altered PG synthesis [12], represent the complexity of specifically identify the different conditions in patients. For instance, *BPNT2* gene mutations have been detected in two patients previously described as Catel-Manzke syndrome [77].

### 3.2. Skeletal Dysplasias Linked to Proteins Involved in GAG Sulfation

*Spondyloepiphyseal dysplasia with congenital joint dislocations, CHST3 type* (SEDCJD, MIM 143095), also named *recessive Larsen syndrome*, is due to biallelic variants in the *CHST3* gene, encoding for carbohydrate sulfotransferase-3, also known as chondroitin 6-sulfotransferase 1 (C6ST-1). Individuals with CHST3 deficiency have dislocation of the knees and/or hips at birth, clubfoot, elbow joint dysplasia with subluxation and limited extension, short stature, and progressive kyphosis developing in late childhood with marked flattening of intervertebral spaces [78]. Since patients affected by this disorder show knee dislocations at birth, the disorder is initially recognised as *Larsen syndrome* (MIM 245600). During childhood, features of spondyloepiphyseal dysplasia become more apparent and the dislocations improve, both spontaneously and with surgical treatment, causing arthritis of the hips and spine with intervertebral disc degeneration, rigid kyphoscoliosis and trunk shortening by late childhood. At this stage, the clinical features are those previously identified as the *Spondyloepiphyseal dysplasia, Omani type* [79]. In conclusion disorders previously designated as *Spondyloepiphyseal dysplasia, Omani type*, *Humerospinal dysostosis* and *recessive Larsen syndrome* represent different age-related descriptions of the same condition. The clinical spectrum underlies the wide mutational spectrum in the *CHST3* gene [78,79,80,81,82]; the gene defects lead to functional impairment of C6ST1 that catalyzes sulfate transfer from PAPS to C6 of N-acetylgalactosamine (GalNAc) residues. This biochemical defect is confirmed by the almost complete depletion of 6-O-sulfated GalNAc residues in CS chains from patients’ fibroblasts and urine [79,80,81]. In some patients, cardiac involvement with systolic murmur, and mitral, tricuspid and aortic regurgitation has been reported [82,83,84]. This issue has not been well studied, but it seems likely that involvement of the cardiac connective tissue may be a genuine, if not constant, feature of CHST3 deficiency, and more attention should be payed to this aspect in the future.

*Ehlers-Danlos syndrome (EDS) musculocontractural type 1* (EDSMC1, MIM 601776)*,* formerly *EDS type VIB* or *adducted thumb-clubfoot syndrome,* include distinctive craniofacial dysmorphism, congenital contractures of thumbs and fingers, clubfeet, severe kyphoscoliosis, muscular hypotonia, hyperextensible thin skin with easy bruisability and atrophic scarring, wrinkled palms, joint hypermobility, atrial septal defects and ocular involvement [85,86]. The causative gene of this disorder is *CHST14* encoding for carbohydrate sulfotransferase-14 (also named dermatan-4-sulfotransferase-1, D4ST1), that catalyzes the 4-O-sulfation of GalNAc residues in dermatan sulfate (DS) [85,86,87]. In patients’ fibroblasts, reduced 4-O-sulfation in GalNAc residues of DS chains, decreased DS and increased CS chain synthesis was demonstrated. The CS increased synthesis is due to the conversion of DS to CS, since the lack of 4-O-sulfation in GalNAc residues allows the reverse epimerization of iduronic acid present in DS chains into glucuronic acid [87]. Thus, decorin DS chains are completely replaced by CS chains leading to reduction of GAG chain flexibility, that in turn affects collagen fibril assembly [88,89]. The presence of congenital contractures in a disorder called “Ehlers-Danlos syndrome” appears to be inappropriate, but the important role of PGs in the development of the skeletal-muscular function unit may account for the phenotype. 

*Osteochondrodysplasia, brachydactyly and overlapping malformed digits* (OCBMD, MIM 618167) has been recently described in a Pakistani family and it is characterised by bilateral and symmetrical skeletal defects affecting mainly the limbs and by hand and foot malformations such as short and adducted thumbs, overriding fingers and broad halluces. Moreover, scoliosis, dislocated patellae and fibulae, pectus excavatum and mild osteoarthritis have been reported. In this family a deletion in the *CHST11* gene encoding for carbohydrate sulfotransferase 11 has been detected [90]. The enzyme also known as chondroitin-4-sulfotrasferase 1 (C4ST1) is a Golgi sulfotransferase responsible for sulfate transfer to C4 of the GalNAc residues in CS chain. Three years earlier, a deletion spanning exon 2 of *CHST11* gene and embedded microRNA MIR3922 was reported in a woman with short stature, brachydactyly with disproportionately short index finger and congenital malformations of hand and feet digits and malignant lymphoproliferative disease [91]. Since combined skeletal phenotype and lymphoproliferative diseases are rare, the involvement in tumour progression of CHST11 is not clear, but it is likely that concomitant loss of MIR3922 or additional genetic and environmental factors may be required [91]. The *Chst11* knock-out mouse confirms the involvement of *CHST11* gene in the chondrodysplasia phenotype [92]. Indeed, homozygous mutant mice die within hours after birth and show severe chondrodysplasia restricted to endochondral bones. Detailed analysis of the cartilage growth plate demonstrated that loss of C4st1 disturbs the balance of CS and causes abnormal CS localization leading to robust up-regulation of transforming growth factor β (TGF-β) signalling with concomitant down-regulation of bone morphogenetic protein (BMP) signalling. These defects result in abnormal chondrocyte differentiation and orientation within the growth plate causing severe disturbances in growth plate morphogenesis [92].

The skeletal disorders which have been discussed in this session are summarized in Table 1.

## 4. Alterations of Extracellular Matrix and Cell Homeostasis Due to Defects in PG Sulfation

PGs are fundamental components of ECMs and affect the biochemical, physical, and mechanical properties of tissues. In particular, PGs are the most abundant ECM proteins in cartilage and they play a pivotal role in the maintenance of cartilage homeostasis. Recent studies underline how altered PG synthesis affects cartilage structure and development, leading to disorders that primarily affect the skeleton [12]. 

Defects in the metabolism of sulfate and mutations in genes encoding for enzymes and transporters involved in the sulfate activation pathway or in sulfotransferase reactions cause altered PG sulfation, affecting PG synthesis and the cartilage ECM properties. The first evidence of impaired sulfate metabolism due to reduced sulfate uptake, decreased PAPS synthesis, or reduced sulfotransferase activity is the synthesis of undersulfated PGs. This condition may affect cartilage homeostasis at different levels impairing the synthesis of whole GAG chains, altering the ECM structure, or the cell behavior. 

In DS biosynthesis, the 4-O-sulfation of GalNAc residue is important to prevent the reconversion of DS chain in CS. CHST14 sulfotransferase impairment reduces the sulfation of GAG chains leading to GAG biosynthetic defects as demonstrated by the complete replacement of DS chain with CS on decorin core protein in fibroblasts from patients affected by EDSMC1 [86,87,88].

In the extracellular environment, PGs are fundamental for interactions with ions, small molecules and proteins and for the organisation of the complex ECM network. For instance, the flexible DS chain of decorin and biglycan allows the correct formation of collagen fibres. TEM analysis of a healthy skin sample shows that the majority of GAG chains are curved and in close contact with the outer of collagen fibres. Conversely, in skin biopsies of patients affected by EDSMC1, collagen fibres are dispersed and GAG chains appear to be straight since DS is converted to CS [86,88,89]. In the bone trabeculae of the animal model of diastrophic dysplasia, the dtd mouse, collagen fibres present a smaller diameter and are less organised in the matrix compared with wild type animals. The undersulfation of CS-PGs, due to impairment of SLC26A2 transporter, could cause defects in collagen fibres organisation leading to an altered bone matrix [96]. 

PGs in ECM and on the cell membrane interact also with signalling molecules through the high negative charge of their GAG chains. Among GAGs, HS is mainly present on the cell surface and it works as co-receptor binding growth factors and making them closer to their receptors. On the other hand, CS in the ECM binds growth factors protecting them from proteases and regulating their diffusion in the matrix. For instance, Ihh signalling, a fundamental pathway for cartilage development, relies on sulfated PGs for its activity as confirmed by the altered Ihh distribution in cartilage ECM of animal models with *PAPPS2* and *SLC26A2* mutations, that causes reduced chondrocyte proliferation [68,97]. Moreover, alteration in Ihh distribution and expression of its downstream targets has been observed also in the *Impad1* knock-out mouse confirming the important link between sulfated PGs and Ihh [58]. Defects in PG sulfation disrupt not only Ihh signalling, but may perturb also other signalling pathways important for regulation of chondrocyte proliferation and differentiation. The downstream effector of BMP signalling is reduced, while the TGF-β effector is increased in growth plate chondrocytes of *Chst11* knock-out mice demonstrating an altered balance between the BMP and TGF-β pathway when PG sulfation is impaired [92].

## 5. Conclusions and Perspectives

Over the last few years, there have been significant advances in the skeletal dysplasia field leading to the identification of the underlying genetic defects in more than 400 different skeletal disorders [15]. The above synopsis highlights the complexity of skeletal defects caused by mutations in genes encoding for enzymes and transporters involved in sulfate metabolism. Progress in this field has been allowed by next-generation genomic technologies, that are a first-line diagnostic resource. In this complex scenario, patient derived biopsies, cell cultures, and animal models are fundamental to investigate the pathogenesis and to analyze new aspects of the role of GAG in connective tissue biology. 

Despite the great step forward in the identification of causative genes, genotype-phenotype correlations are lacking and we are still far from a comprehensive view of the disease molecular mechanisms. First, it is unclear how the tissue specificity and the redundancy of genes can determine the phenotype. Defects in PG sulfation mainly affect cartilage and bone, but other tissues can be involved as cardiac tissue in SEDCJD [82,84] or lymphoid tissue leading to tumour progression in OCBMD [91]. The involvement of different tissues and its implications on the disease phenotype should be carefully studied in the future. Moreover, mutations in different genes cause skeletal dysplasias with overlapping features that may be wrongly diagnosed as occurs in condrodysplasia with joint dislocation, gPAPP type, Catel-Manzke syndrome and Desbuquois dysplasia type 1. Nowadays we cannot provide a full explanation why some classes of sulfated PGs are more affected by enzyme deficiency than others. Even if the GAGs role depends on their physicochemical properties, it is difficult to molecularly dissect the function of sulfated GAGs when they interact in the complex ECM network. Lastly, the variability in the clinical phenotypes caused by mutations in the same gene suggests that also environmental and epigenetic factors might play a role. 

A deep understanding of the molecular mechanisms of these disorders is crucial to ultimately pave the way for innovative therapies. Recently, significant advances in the development of therapeutic approaches for some disorders have been achieved with the aim of alleviating, preventing or modifying the disease progression [70,98,99,100,101]. This progress has been made thank to deep phenotyping of in vitro and/or in vivo models that has allowed the identification of new molecules or already existing ones through a drug repositioning approach. Moreover, molecular studies in different disorders could lead to the identification of common pathogenic mechanisms resulting in common therapeutic strategies that might be targeted to a range of individual phenotypes.

## Figures and Tables

**Table 1:** Skeletal dysplasias caused by defects in sulfate metabolism.

Pathology	MIM/Inheritance	Causative Gene	Protein Product and Function	Biochemical Phenotype	References
Achondrogenesis type 1B (ACG1B)	600972/AR	*SLC26A2*	Sulfate/chloride antiporter present on cell membrane.	Severe cartilage PG undersulfation; reduced sulfate uptake in fibroblasts.	[60,65,66,71,93]
Atelosteogenesis type 2 (AO2)	256050/AR	*SLC26A2*	Sulfate/chloride antiporter present on cell membrane.	Severe cartilage PG undersulfation; reduced sulfate uptake in fibroblasts.	[60,65,66,94]
Diastrophic dysplasia (DTD)	222600/AR	*SLC26A2*	Sulfate/chloride antiporter present on cell membrane.	Cartilage PG undersulfation; reduced sulfate uptake in fibroblasts; in mice altered sulfate uptake in chondrocytes, and osteoblasts; altered Ihh signaling; reduced chondrocytes proliferation.	[34,60,65,66,67,68,69,70]
Recessive multiple epiphyseal dysplasia (EDM4)	226900/AR	*SLC26A2*	Sulfate/chloride antiporter present on cell membrane.	Reduced sulfate uptake.	[60,65,95]
Spondyloepimetaphyseal dysplasia, SEMD, Pakistani type or Brachyolmia type 4 (BCYM4)	612847/AR	*PAPSS2*	PAPS synthetase 2, enzyme that synthesizes the universal sulfate donor (PAPS).	Macromolecular undersulfation; signs of androgen excess (in a minority of patients); very low DHEAS levels and increased androgen levels (in one patient).	[40,42,72,73,76]
Brachyolmia type 1 (includes Hobaek form and Toledo form, BCYM1A and 1B respectively)	271530/AR 271630/AR	*PAPSS2*	PAPS synthetase 2, enzyme that synthesizes the universal sulfate donor (PAPS).	Undersulfation of CS; low activity of PAPS-CS sulfotransferase; signs of androgen excess (in a minority of patients).	[73,74,75,76]
Chondrodysplasia with joint dislocations, gPAPP type	614078/AR	*BPNT2*	Golgi resident PAP phosphatase, enzyme that hydrolyzes PAP to AMP and phosphate.	In mice impaired CS and HS sulfation.	[56,57,58,77]
Spondyloepiphyseal dysplasia with congenital joint dislocations (SEDCJD or SED Omani type)	143095/AR	*CHST3*	Carbohydrate sulfotransferase-3, enzyme that transfers sulfate to GalNAc residues of CS.	Depletion of 6-O-sulfated GalNAc residues in CS chains in fibroblasts and urine.	[78,79,80,81,82,83,84]
Ehlers-Danlos syndrome musculocontractural type 1 (EDSMC1)	601776/AR	*CHST14*	Carbohydrate sulfotransferase-14, enzyme that transfers sulfate to GalNAc residues of DS.	Reduction of 4-O-sulfation in GalNAc residues in DS chains; decrease of DS and increase of CS chain synthesis.	[85,86,87,88,89]
Osteochondrodysplasia, brachydactyly and overlapping malformed digits (OCBMD)	618167/AR	*CHST11*	Carbohydrate sulfotransferase-11, enzyme that transfers sulfate to GalNAc residues of CS.	In mice abnormal CS localization; strong up-regulation of TGF-β signaling and down-regulation of BMP signaling; altered morphology of the growth plate.	[90,91,92]

## Abbreviations

ACG1B	achondrogenesis type 1B
AO2	atelosteogenesis type 2
APS	adenosine 5′-phosphosulfate
BCYM1A	brachyolmia type 1 Hobaek form
BCYM1B	brachyolmia type 1 Toledo form
BCYM4	brachyolmia type 4
BPNT2	3′(2′) 5′-bisphosphate nucleotidase 2
C4ST	chondroitin-4-O-sulfotransferase
C6ST	chondroitin-6-O-sulfotransferase
CS	chondroitin sulfate
D4ST	dermatan-4-O-sulfotransferase
DHEA	dehydroepiandrosterone
DHEAS	dehydroepiandrosterone sulfate ester
DS	dermatan sulfate
DTD	diastrophic dysplasia
DTDST	diastrophic dysplasia sulfate transporter
ECM	extracellular matrix
EDM4	recessive multiple epiphyseal dysplasia
EDSMC1	Ehlers-Danlos syndrome musculocontractural type 1
GAG	glycosaminoglycan
GalNAc	N-acetylgalactosamine
gPAPP	Golgi resident phosphoadenosine phosphate phosphatase
HS	heparan sulfate
IMPAD1	inositol monophosphatase domain-containing protein 1
OCBMD	osteochondrodysplasia brachydactyly and overlapping malformed digits
PAP	phosphoadenosine phosphate
PAPS	3′-phosphoadenosine 5′-phosphosulfate
PAPSS	PAPS synthetase
PAPST	PAPS transporter
PG	proteoglycan
SEDCJD	spondyloepiphyseal dysplasia with congenital joint dislocations
ST	carbohydrate sulfotransferase
SULT	cytosolic sulfotransferase
TPST	tyrosylprotein sulfotransferase

## References

[1] Iozzo R.V. (1998). Matrix proteoglycans: From molecular design to cellular function. Annu. Rev. Biochem..

[2] Honke K., Taniguchi N. (2002). Sulfotransferases and sulfated oligosaccharides. Med. Res. Rev..

[3] Bowman K.G., Bertozzi C.R. (1999). Carbohydrate sulfotransferases: Mediators of extracellular communication. Chem. Biol..

[4] Gallagher J.T. (1994). Heparan sulphates as membrane receptors for the fibroblast growth factors. Eur. J. Clin. Chem. Clin. Biochem..

[5] Pacifici G.M. (2005). Sulfation of drugs and hormones in mid-gestation human fetus. Early Hum. Dev..

[6] Farooqui A.A., Horrocks L.A. (1985). On the role of sulfolipids in mammalian metabolism. Mol. Cell. Biochem..

[7] Stone M.J., Chuang S., Hou X., Shoham M., Zhu J.Z. (2009). Tyrosine sulfation: An increasingly recognised post-translational modification of secreted proteins. Nat. Biotechnol..

[8] Richard K., Hume R., Kaptein E., Stanley E.L., Visser T.J., Coughtrie M.W. (2001). Sulfation of thyroid hormone and dopamine during human development: Ontogeny of phenol sulfotransferases and arylsulfatase in liver, lung, and brain. J. Clin. Endocrinol. Metab..

[9] Aksoy I.A., Otterness D.M., Weinshilboum R.M. (1993). Cholesterol sulfation in human liver. Catalysis by dehydroepiandrosterone sulfotransferase. Drug Metab. Dispos..

[10] Alnouti Y. (2009). Bile Acid sulfation: A pathway of bile acid elimination and detoxification. Toxicol. Sci..

[11] Falany C.N., Wheeler J., Oh T.S., Falany J.L. (1994). Steroid sulfation by expressed human cytosolic sulfotransferases. J. Steroid Biochem. Mol. Biol..

[12] Paganini C., Costantini R., Superti-Furga A., Rossi A. (2019). Bone and connective tissue disorders caused by defects in glycosaminoglycan biosynthesis: A panoramic view. FEBS J..

[13] Krakow D., Rimoin D.L. (2010). The skeletal dysplasias. Genet. Med..

[14] Geister K.A., Camper S.A. (2015). Advances in Skeletal Dysplasia Genetics. Annu. Rev. Genom. Hum. Genet..

[15] Mortier G.R., Cohn D.H., Cormier-Daire V., Hall C., Krakow D., Mundlos S., Nishimura G., Robertson S., Sangiorgi L., Savarirayan R. (2019). Nosology and classification of genetic skeletal disorders: 2019 revision. Am. J. Med. Genet. A.

[16] Klaassen C.D., Boles J.W. (1997). Sulfation and sulfotransferases 5: The importance of 3’- phosphoadenosine 5’-phosphosulfate (PAPS) in the regulation of sulfation. FASEB J..

[17] Leyh T.S. (1993). The physical biochemistry and molecular genetics of sulfate activation. Crit. Rev. Biochem. Mol. Biol..

[18] Lipmann F. (1958). Biological sulfate activation and transfer. Science.

[19] Farooqui A.A. (1980). 3’-phosphoadenosine 5’-phosphosulphate metabolism in mammalian tissues. Int. J. Biochem..

[20] Venkatachalam K.V. (2003). Human 3’-phosphoadenosine 5’-phosphosulfate (PAPS) synthase: Biochemistry, molecular biology and genetic deficiency. Iubmb Life.

[21] Langford R., Hurrion E., Dawson P.A. (2017). Genetics and pathophysiology of mammalian sulfate biology. J. Genet. Genom..

[22] Dawson P.A. (2011). Sulfate in fetal development. Semin. Cell Dev. Biol..

[23] Dawson P.A., Richard K., Perkins A., Zhang Z., Simmons D.G. (2017). Review: Nutrient sulfate supply from mother to fetus: Placental adaptive responses during human and animal gestation. Placenta.

[24] Cole D.E., Scriver C.R. (1980). Age-dependent serum sulfate levels in children and adolescents. Clin. Chim. Acta.

[25] Cole D.E., Baldwin L.S., Stirk L.J. (1985). Increased serum sulfate in pregnancy: Relationship to gestational age. Clin. Chem..

[26] Bradley H., Gough A., Sokhi R.S., Hassell A., Waring R., Emery P. (1994). Sulfate metabolism is abnormal in patients with rheumatoid arthritis. Confirmation by in vivo biochemical findings. J. Rheumatol..

[27] Jennings M.L. (1976). Proton fluxes associated with erythrocyte membrane anion exchange. J. Membr. Biol..

[28] Lötscher M., Custer M., Quabius E.S., Kaissling B., Murer H., Biber J. (1996). Immunolocalization of Na/SO4-cotransport (NaSi-1) in rat kidney. Pflug. Arch..

[29] Simmons D.G., Rakoczy J., Jefferis J., Lourie R., McIntyre H.D., Dawson P.A. (2013). Human placental sulfate transporter mRNA profiling from term pregnancies identifies abundant SLC13A4 in syncytiotrophoblasts and SLC26A2 in cytotrophoblasts. Placenta.

[30] Dawson P.A., Markovich D. (2005). Pathogenetics of the human SLC26 transporters. Curr. Med. Chem..

[31] Alper S.L., Sharma A.K. (2013). The SLC26 gene family of anion transporters and channels. Mol. Asp. Med..

[32] Markovich D. (2014). Na+-sulfate cotransporter SLC13A1. Pflug. Arch..

[33] Zhang Z., Aung Z.T., Simmons D.G., Dawson P.A. (2017). Molecular analysis of sequence and splice variants of the human SLC13A4 sulfate transporter. Mol. Genet. Metab..

[34] Hästbacka J., de la Chapelle A., Mahtani M.M., Clines G., Reeve Daly M.P., Daly M., Hamilton B.A., Kusumi K., Trivedi B., Weaver A. (1994). The diastrophic dysplasia gene encodes a novel sulfate transporter: Positional cloning by fine-structure linkage disequilibrium mapping. Cell.

[35] Dawson J.R., Norbeck K., Moldeus P. (1983). The effectiveness of different sulfate precursors in supporting extrahepatic sulfate conjugation. Biochem. Pharm..

[36] Elgavish A., Meezan E. (1991). Sulfation by human lung fibroblasts: SO4(2-) and sulfur- containing amino acids as sources for macromolecular sulfation. Am. J. Physiol..

[37] Rome L.H., Hill D.F. (1986). Lysosomal degradation of glycoproteins and glycosaminoglycans. Efflux and recycling of sulphate and N-acetylhexosamines. Biochem. J..

[38] Lyle S., Stanczak J., Ng K., Schwartz N.B. (1994). Rat chondrosarcoma ATP sulfurylase and adenosine 5’-phosphosulfate kinase reside on a single bifunctional protein. Biochemistry.

[39] Xu Z.H., Otterness D.M., Freimuth R.R., Carlini E.J., Wood T.C., Mitchell S., Moon E., Kim U.J., Xu J.P., Siciliano M.J. (2000). Human 3’-phosphoadenosine 5’-phosphosulfate synthetase 1 (PAPSS1) and PAPSS2: Gene cloning, characterization and chromosomal localization. Biochem. Biophys. Res. Commun..

[40] ul Haque M.F., King L.M., Krakow D., Cantor R.M., Rusiniak M.E., Swank R.T., Superti-Furga A., Haque S., Abbas H., Ahmad W. (1998). Mutations in orthologous genes in human spondyloepimetaphyseal dysplasia and the brachymorphic mouse. Nat. Genet..

[41] Kurima K., Warman M.L., Krishnan S., Domowicz M., Krueger R.C., Deyrup A., Schwartz N.B. (1998). A member of a family of sulfate-activating enzymes causes murine brachymorphism. Proc. Natl. Acad. Sci. USA.

[42] Noordam C., Dhir V., McNelis J.C., Schlereth F., Hanley N.A., Krone N., Smeitink J.A., Smeets R., Sweep F.C., Claahsen-van der Grinten H.L. (2009). Inactivating PAPSS2 mutations in a patient with premature pubarche. N. Engl. J. Med..

[43] Strott C.A. (2002). Sulfonation and molecular action. Endocr. Rev..

[44] Fuda H., Shimizu C., Lee Y.C., Akita H., Strott C.A. (2002). Characterization and expression of human bifunctional 3’-phosphoadenosine 5’-phosphosulphate synthase isoforms. Biochem. J..

[45] Schroder E., Gebel L., Eremeev A.A., Morgner J., Grum D., Knauer S.K., Bayer P., Mueller J.W. (2012). Human PAPS Synthase Isoforms Are Dynamically Regulated Enzymes with Access to Nucleus and Cytoplasm. PLoS ONE.

[46] Kamiyama S., Sasaki N., Goda E., Ui-Tei K., Saigo K., Narimatsu H., Jigami Y., Kannagi R., Irimura T., Nishihara S. (2006). Molecular cloning and characterization of a novel 3’-phosphoadenosine 5’-phosphosulfate transporter, PAPST2. J. Biol. Chem..

[47] Kamiyama S., Suda T., Ueda R., Suzuki M., Okubo R., Kikuchi N., Chiba Y., Goto S., Toyoda H., Saigo K. (2003). Molecular cloning and identification of 3’-phosphoadenosine 5’-phosphosulfate transporter. J. Biol. Chem..

[48] Günal S., Hardman R., Kopriva S., Mueller J.W. (2019). Sulfation pathways from red to green. J. Biol. Chem..

[49] Parker J.L., Newstead S. (2019). Gateway to the Golgi: Molecular mechanisms of nucleotide sugar transporters. Curr. Opin. Struct. Biol..

[50] Wiweger M.I., Avramut C.M., de Andrea C.E., Prins F.A., Koster A.J., Ravelli R.B., Hogendoorn P.C. (2011). Cartilage ultrastructure in proteoglycan-deficient zebrafish mutants brings to light new candidate genes for human skeletal disorders. J. Pathol..

[51] Bojarova P., Williams S.J. (2008). Sulfotransferases, sulfatases and formylglycine-generating enzymes: A sulfation fascination. Curr. Opin. Chem. Biol..

[52] Coughtrie M.W.H. (2016). Function and organization of the human cytosolic sulfotransferase (SULT) family. Chem. Biol. Interact..

[53] Petrotchenko E.V., Pedersen L.C., Borchers C.H., Tomer K.B., Negishi M. (2001). The dimerization motif of cytosolic sulfotransferases. FEBS Lett..

[54] Hartmann-Fatu C., Trusch F., Moll C.N., Michin I., Hassinen A., Kellokumpu S., Bayer P. (2015). Heterodimers of tyrosylprotein sulfotransferases suggest existence of a higher organization level of transferases in the membrane of the trans-Golgi apparatus. J. Mol. Biol..

[55] Leung A.W., Backstrom I., Bally M.B. (2016). Sulfonation, an underexploited area: From skeletal development to infectious diseases and cancer. Oncotarget.

[56] Vissers L.E., Lausch E., Unger S., Campos-Xavier A.B., Gilissen C., Rossi A., Del Rosario M., Venselaar H., Knoll U., Nampoothiri S. (2011). Chondrodysplasia and abnormal joint development associated with mutations in IMPAD1, encoding the Golgi-resident nucleotide phosphatase, gPAPP. Am. J. Hum. Genet..

[57] Frederick J.P., Tafari A.T., Wu S.M., Megosh L.C., Chiou S.T., Irving R.P., York J.D. (2008). A role for a lithium-inhibited Golgi nucleotidase in skeletal development and sulfation. Proc. Natl. Acad. Sci. USA.

[58] Sohaskey M.L., Yu J., Diaz M.A., Plaas A.H., Harland R.M. (2008). JAWS coordinates chondrogenesis and synovial joint positioning. Development.

[59] Diez-Roux G., Ballabio A. (2005). Sulfatases and human disease. Annu. Rev. Genom. Hum. Genet..

[60] Rossi A., Superti-Furga A. (2001). Mutations in the diastrophic dysplasia sulfate transporter (DTDST) gene (SLC26A2): 22 Novel mutations, mutation review, associated skeletal phenotypes, and diagnostic relevance. Hum. Mutat..

[61] De la Chapelle A., Maroteaux P., Havu N., Granroth G. (1972). A rare lethal bone dysplasia with recessive autosomic transmission. Arch. Fr. Pediatr..

[62] McAlister W.H., Crane J.P., Bucy R.P., Craig R.B. (1985). A new neonatal short limbed dwarfism. Skelet. Radiol..

[63] Bonafe L., Hastbacka J., de la Chapelle A., Campos-Xavier A.B., Chiesa C., Forlino A., Superti-Furga A., Rossi A. (2008). A novel mutation in the sulfate transporter gene SLC26A2 (DTDST) specific to the Finnish population causes de la Chapelle dysplasia. J. Med. Genet..

[64] Rossi A., Bonaventure J., Delezoide A.L., SupertiFurga A., Cetta G. (1997). Undersulfation of cartilage proteoglycans ex vivo and increased contribution of amino acid sulfur to sulfation in vitro in McAlister dysplasia atelosteogenesis type 2. Eur. J. Biochem..

[65] Karniski L.P. (2001). Mutations in the diastrophic dysplasia sulfate transporter (DTDST) gene: Correlation between sulfate transport activity and chondrodysplasia phenotype. Hum. Mol. Genet..

[66] Rossi A., Kaitila I., Wilcox W.R., Rimoin D.L., Steinmann B., Cetta G., Superti-Furga A. (1998). Proteoglycan sulfation in cartilage and cell cultures from patients with sulfate transporter chondrodysplasias: Relationship to clinical severity and indications on the role of intracellular sulfate production. Matrix Biol..

[67] Forlino A., Piazza R., Tiveron C., Della Torre S., Tatangelo L., Bonafe L., Gualeni B., Romano A., Pecora F., Superti-Furga A. (2005). A diastrophic dysplasia sulfate transporter (SLC26A2) mutant mouse: Morphological and biochemical characterization of the resulting chondrodysplasia phenotype. Hum. Mol. Genet..

[68] Gualeni B., Facchini M., De Leonardis F., Tenni R., Cetta G., Viola M., Passi A., Superti-Furga A., Forlino A., Rossi A. (2010). Defective proteoglycan sulfation of the growth plate zones causes reduced chondrocyte proliferation via an altered Indian hedgehog signalling. Matrix Biol..

[69] De Leonardis F., Monti L., Gualeni B., Tenni R., Forlino A., Rossi A. (2014). Altered signaling in the G1 phase deregulates chondrocyte growth in a mouse model with proteoglycan undersulfation. J. Cell. Biochem..

[70] Monti L., Paganini C., Lecci S., De Leonardis F., Hay E., Cohen-Solal M., Villani S., Superti-Furga A., Tenni R., Forlino A. (2015). N-acetylcysteine treatment ameliorates the skeletal phenotype of a mouse model of diastrophic dysplasia. Hum. Mol. Genet..

[71] Zheng C., Lin X., Xu X., Wang C., Zhou J., Gao B., Fan J., Lu W., Hu Y., Jie Q. (2019). Suppressing UPR-dependent overactivation of FGFR3 signaling ameliorates SLC26A2-deficient chondrodysplasias. EBioMedicine.

[72] Ahmad M., Haque M.F., Ahmad W., Abbas H., Haque S., Krakow D., Rimoin D.L., Lachman R.S., Cohn D.H. (1998). Distinct, autosomal recessive form of spondyloepimetaphyseal dysplasia segregating in an inbred Pakistani kindred. Am. J. Med. Genet..

[73] Miyake N., Elcioglu N.H., Iida A., Isguven P., Dai J., Murakami N., Takamura K., Cho T.J., Kim O.H., Hasegawa T. (2012). PAPSS2 mutations cause autosomal recessive brachyolmia. J. Med. Genet..

[74] Toledo S.P., Mourao P.A., Lamego C., Alves C.A., Dietrich C.P., Assis L.M., Mattar E. (1978). Recessively inherited, late onset spondylar dysplasia and peripheral corneal opacity with anomalies in urinary mucopolysaccharides: A possible error of chondroitin-6-sulfate synthesis. Am. J. Med. Genet..

[75] Mourao P.A., Kato S., Donnelly P.V. (1981). Spondyloepiphyseal dysplasia, chondroitin sulfate type: A possible defect of PAPS--chondroitin sulfate sulfotransferase in humans. Biochem. Biophys. Res. Commun..

[76] Iida A., Simsek-Kiper P.O., Mizumoto S., Hoshino T., Elcioglu N., Horemuzova E., Geiberger S., Yesil G., Kayserili H., Utine G.E. (2013). Clinical and radiographic features of the autosomal recessive form of brachyolmia caused by PAPSS2 mutations. Hum. Mutat..

[77] Nizon M., Alanay Y., Tuysuz B., Kiper P.O., Genevieve D., Sillence D., Huber C., Munnich A., Cormier-Daire V. (2012). IMPAD1 mutations in two Catel-Manzke like patients. Am. J. Med. Genet. A.

[78] Unger S., Lausch E., Rossi A., Megarbane A., Sillence D., Alcausin M., Aytes A., Mendoza-Londono R., Nampoothiri S., Afroze B. (2010). Phenotypic features of carbohydrate sulfotransferase 3 (CHST3) deficiency in 24 patients: Congenital dislocations and vertebral changes as principal diagnostic features. Am. J. Med. Genet. A.

[79] Thiele H., Sakano M., Kitagawa H., Sugahara K., Rajab A., Hohne W., Ritter H., Leschik G., Nurnberg P., Mundlos S. (2004). Loss of chondroitin 6-O-sulfotransferase-1 function results in severe human chondrodysplasia with progressive spinal involvement. Proc. Natl. Acad. Sci. USA.

[80] Hermanns P., Unger S., Rossi A., Perez-Aytes A., Cortina H., Bonafe L., Boccone L., Setzu V., Dutoit M., Sangiorgi L. (2008). Congenital joint dislocations caused by carbohydrate sulfotransferase 3 deficiency in recessive Larsen syndrome and humero-spinal dysostosis. Am. J. Hum. Genet..

[81] van Roij M.H., Mizumoto S., Yamada S., Morgan T., Tan-Sindhunata M.B., Meijers-Heijboer H., Verbeke J.I., Markie D., Sugahara K., Robertson S.P. (2008). Spondyloepiphyseal dysplasia, Omani type: Further definition of the phenotype. Am. J. Med. Genet. A.

[82] Tuysuz B., Mizumoto S., Sugahara K., Celebi A., Mundlos S., Turkmen S. (2009). Omani-type spondyloepiphyseal dysplasia with cardiac involvement caused by a missense mutation in CHST3. Clin. Genet..

[83] Kozlowski K.S., Celermajer J.M., Tink A.R. (1974). Humero-spinal dysostosis with congenital heart disease. Am. J. Dis. Child..

[84] Hall B.D. (1997). Humero-spinal dysostosis: Report of the fourth case with emphasis on generalized skeletal involvement, abnormal craniofacial features, and mitral valve thickening. J. Pediatr. Orthop. B.

[85] Malfait F., Syx D., Vlummens P., Symoens S., Nampoothiri S., Hermanns-Le T., Van Laer L., De Paepe A. (2010). Musculocontractural Ehlers-Danlos Syndrome (former EDS type VIB) and adducted thumb clubfoot syndrome (ATCS) represent a single clinical entity caused by mutations in the dermatan-4-sulfotransferase 1 encoding CHST14 gene. Hum. Mutat..

[86] Janecke A.R., Li B., Boehm M., Krabichler B., Rohrbach M., Muller T., Fuchs I., Golas G., Katagiri Y., Ziegler S.G. (2016). The phenotype of the musculocontractural type of Ehlers-Danlos syndrome due to CHST14 mutations. Am. J. Med. Genet. A.

[87] Dundar M., Muller T., Zhang Q., Pan J., Steinmann B., Vodopiutz J., Gruber R., Sonoda T., Krabichler B., Utermann G. (2009). Loss of dermatan-4-sulfotransferase 1 function results in adducted thumb-clubfoot syndrome. Am. J. Hum. Genet..

[88] Miyake N., Kosho T., Mizumoto S., Furuichi T., Hatamochi A., Nagashima Y., Arai E., Takahashi K., Kawamura R., Wakui K. (2010). Loss-of-function mutations of CHST14 in a new type of Ehlers-Danlos syndrome. Hum. Mutat..

[89] Hirose T., Takahashi N., Tangkawattana P., Minaguchi J., Mizumoto S., Yamada S., Miyake N., Hayashi S., Hatamochi A., Nakayama J. (2018). Structural alteration of glycosaminoglycan side chains and spatial disorganization of collagen networks in the skin of patients with mcEDS-CHST14. Biochim. Biophys. Acta Gen. Subj..

[90] Shabbir R.M.K., Nalbant G., Ahmad N., Malik S., Tolun A. (2018). Homozygous *CHST11* mutation in chondrodysplasia, brachydactyly, overriding digits, clino-symphalangism and synpolydactyly. J. Med. Genet..

[91] Chopra S.S., Leshchiner I., Duzkale H., McLaughlin H., Giovanni M., Zhang C., Stitziel N., Fingeroth J., Joyce R.M., Lebo M. (2015). Inherited CHST11/MIR3922 deletion is associated with a novel recessive syndrome presenting with skeletal malformation and malignant lymphoproliferative disease. Mol. Genet. Genom. Med..

[92] Kluppel M., Wight T.N., Chan C., Hinek A., Wrana J.L. (2005). Maintenance of chondroitin sulfation balance by chondroitin-4-sulfotransferase 1 is required for chondrocyte development and growth factor signaling during cartilage morphogenesis. Development.

[93] SupertiFurga A., Hastbacka J., Wilcox W.R., Cohn D.H., vanderHarten H.J., Rossi A., Blau N., Rimoin D.L., Steinmann B., Lander E.S. (1996). Achondrogenesis type IB is caused by mutations in the diastrophic dysplasia sulphate transporter gene. Nat. Genet..

[94] Hästbacka J., Superti-Furga A., Wilcox W.R., Rimoin D.L., Cohn D.H., Lander E.S. (1996). Atelosteogenesis type II is caused by mutations in the diastrophic dysplasia sulfate-transporter gene (DTDST): Evidence for a phenotypic series involving three chondrodysplasias. Am. J. Hum. Genet..

[95] Superti-Furga A., Neumann L., Riebel T., Eich G., Steinmann B., Spranger J., Kunze J. (1999). Recessively inherited multiple epiphyseal dysplasia with normal stature, club foot, and double layered patella caused by a DTDST mutation. J. Med. Genet..

[96] Gualeni B., de Vernejoul M.C., Marty-Morieux C., De Leonardis F., Franchi M., Monti L., Forlino A., Houillier P., Rossi A., Geoffroy V. (2013). Alteration of proteoglycan sulfation affects bone growth and remodeling. Bone.

[97] Cortes M., Baria A.T., Schwartz N.B. (2009). Sulfation of chondroitin sulfate proteoglycans is necessary for proper Indian hedgehog signaling in the developing growth plate. Development.

[98] Besio R., Antonella F. (2015). Treatment options for osteogenesis imperfecta. Expert Opin. Orphan Drugs.

[99] Briggs M.D., Bell P.A., Wright M.J., Pirog K.A. (2015). New therapeutic targets in rare genetic skeletal diseases. Expert Opin. Orphan Drugs.

[100] Ornitz D.M., Legeai-Mallet L. (2017). Achondroplasia: Development, pathogenesis, and therapy. Dev. Dyn..

[101] Marzin P., Cormier-Daire V. (2020). New perspectives on the treatment of skeletal dysplasia. Adv. Endocrinol. Metab..

